# Association Between PM_2.5_ Exposure Level and Primary Open-Angle Glaucoma in Taiwanese Adults: A Nested Case–control Study

**DOI:** 10.3390/ijerph18041714

**Published:** 2021-02-10

**Authors:** Han-Yin Sun, Ci-Wen Luo, Yun-Wei Chiang, Kun-Lin Yeh Yi-Ching Li, Yung-Chung Ho, Shiuan-Shinn Lee, Wen-Ying Chen, Chun-Jung Chen, Yu-Hsiang Kuan

**Affiliations:** 1 Department of Optometry, Chung Shan Medical University, Taichung 40201, Taiwan; hanying@csmu.edu.tw; 2 Department of Ophthalmology, Chung Shan Medical University Hospital, Taichung 40201, Taiwan; 3 Department of Pharmacology, School of Medicine, Chung Shan Medical University, Taichung 40201, Taiwan; kkjj88440@gmail.com (C.-W.L.);; 4 Department of Pharmacy, Chung Shan Medical University Hospital, Taichung 40201, Taiwan; 5 Department of life sciences, National Chung-Hsing University, Taichung 402204, Taiwan; apple3793@gmail.com; 6 Department of Veterinary Medicine, National Chung Hsing University, Taichung 402204, Taiwan; bill05002@gmail.com; 7 School of Medical Applied Chemistry, Chung Shan Medical University, Taichung 40201, Taiwan; ych065@csmu.edu.tw; 8 School of Public Health, Chung Shan Medical University, Taichung 40201, Taiwan; shinn@csmu.edu.tw; 9 Department of Education and Research, Taichung Veterans General Hospital, Taichung 40705, Taiwan; cjchen@vghtc.gov.tw

**Keywords:** PM_2.5_, primary open-angle glaucoma, Taiwanese adults, nested case–control study

## Abstract

Primary open-angle glaucoma (POAG) is the most common type of glaucoma. However, little is known about POAG in adults and exposure to air pollution. The current study aims to investigate whether exposure to particulate matter with a mass median aerodynamic diameter of ≤2.5 μm (PM_2.5_) is associated with POAG diagnosis. Patient data were obtained from the Longitudinal Health Insurance Database 2010 (LHID2010) of Taiwan for the 2008–2013 period. PM_2.5_ concentration data, collected from the Ambient Air Quality Monitoring Network established by the Environmental Protection Administration of Taiwan, were categorized into four groups according to World Health Organization (WHO) exposure standards for PM_2.5_. We estimated the odds ratios (ORs) and 95% CIs for risk factors for POAG with logistic regression. The OR of per WHO standard level increase was 1.193 (95% CI 1.050–1.356). Compared with the normal level, the OR of WHO 2.0 level was 1.668 (95% CI 1.045–2.663, *P* < 0.05). After excluding confounding risk factors for POAG in this study, we determined that increased PM_2.5_ exposure is related to POAG risk (ORs > 1, *P* < 0.05). In this study, PM_2.5_ was an independent factor associated with open-angle glaucoma. Further research is required to better understand the mechanisms connecting PM_2.5_ and open-angle glaucoma.

## 1. Introduction

Particulate matter with a mass median aerodynamic diameter of ≤2.5 μm (PM_2.5_) is typically composed of mixtures of some solid and liquid droplets. The sources of PM_2.5_ are both natural and artificial. Artificial PM_2.5_ generated by road vehicles and industrial concerns is of greater significance than that from natural sources. The World Health Organization’s (WHO’s) air quality guidelines specify minimum concentrations that affect health [1] (10 and 25 μg/m^3^ for long-term and short-term exposure, respectively). Higher air pollution from low- and middle-income countries is responsible for an estimated 5.9 million premature deaths linked to indoor and outdoor every year [2]. Short-term exposure to PM_2.5_ has been associated with premature mortality, increased risk of heart or lung disease, acute and chronic bronchitis, respiratory symptoms, and neurological disease. Long-term exposure to PM_2.5_ results in increased premature death rates in people who have chronic heart or lung diseases.

Epidemiological studies have suggested that pollution is associated with increased risk of central nervous system diseases that affect the cranial nerves (including Alzheimer’s disease and Parkinson’s disease), cardiovascular disease (e.g., stroke and ischemic heart disease), and respiratory system disease (e.g., asthma and acute respiratory infections) [3,4,5]. Experimental data both in vivo and in vitro have demonstrated PM-induced oxidative stress, inflammatory reactions, and neurotransmitter changes that affect brain development and cause pathogenesis of central nervous system diseases [6]. In vitro studies have shown that particles vary significantly in their cytotoxic and inflammatory effects using cultured lung cell models. In animal models [7], chronic exposure to air pollutants has been shown to increase cytokine production in the brain, producing changes in neuronal structure and function, impacts on neurotransmitters, axis dysfunction, neurodegenerative disease, and depression [8,9]. The eye is one of the few organs directly exposed to the external environment. Particulate matter has been reported to promote stress and systemic inflammation in various cells, including corneal cell apoptosis and inflammation [10]. People exposed to high concentrations of pollutants may complain of ocular symptoms such as irritation, dryness, burning, and itching [11,12]. Other studies have reported that conjunctivitis and decreased tear film pH are significantly associated with air pollution [13,14]. Furthermore, an association between retinal vessel disability and national levels of PM_2.5_ has been proposed [15,16].

Glaucoma, a disease that damages the optic nerve, is a leading cause of blindness for people aged >60 years [17]. Glaucoma risk increases with age [18,19], and its prevalence is associated with age-related diseases such as macular degeneration and vascular lesion [18,20]. Rates of glaucoma for people living in urban areas are 1.5 times higher than for those living in rural areas, making air pollution a potential risk factor for the disease [21]. Primary open-angle glaucoma (POAG) is the most common type. However, little is known about the occurrence of POAG in adults and exposure to air pollution. Demonstration of a positive association between air pollution and POAG could suggest a novel risk factor and provide medical evidence for campaigns to reduce particulate air pollutants. In this study, we investigate whether exposure to air pollution, particularly to PM_2.5_, is associated with diagnosis of POAG.

## 2. Materials and Methods

### 2.1. Data Sources

Patient data were obtained from the Longitudinal Health Insurance Database 2010 (LHID2010). This database is a random sample of 1 million people from the National Health Insurance Database (NHIRD), and we established that there were no statistically significant differences in age, gender, distribution of births per year, and average insured amount. NHIRD records information on medical services for >99% of the 23 million Taiwan residents. The database contains information on inpatient and outpatient visits, for which data are coded by clinicians according to the International Classification of Diseases, Ninth Revision, Clinical Modification (ICD-9-CM).

### 2.2. Collection of PM_2.5_ Concentration Data

The Ambient Air Quality Monitoring Network (AQMN) used a tapered element oscillating microbalance (R&P 1400, Rupprecht and Patashnick, New York, NY, US) to measure PM_2.5_ concentration in the atmosphere, and recorded the measured value every hour. This information was provided by the Taiwan Environmental Protection Agency. The study protocol used month as the unit for time measurement, so the monthly average cumulative exposure was used as the metric of patient exposure. First, benchmarks were based on the exposure of the station in the patient’s residence district; if there was no station in the patient’s residence district, the next closest station was used as the benchmark. Second, we excluded missing values of patient exposure according to the following criteria: during the observation period, if the daily observation result contained more than 8 h of missing values, it would be excluded; if the monthly observation result contained more than 10 days of missing values, it would be excluded. The average daily exposure was calculated as the daily exposure divided by 24, and the average monthly exposure was calculated as the average daily exposure multiplied by the number of days in the month. Third, we observed the PM_2.5_ exposure during the five-year period, and the missing values of PM_2.5_ during this period are relatively small [22,23]. Fourth, we used the data provided by AQMN to estimate the monthly average PM_2.5_ concentration, and devised groupings to examine the exposure–response relationship. Data covering the observation period were categorized into four groups according to WHO exposure standards for PM_2.5_: normal level (<25 μg/m^3^ × exposure months); WHO 1.0 level (≥1 to <1.5 × [25 μg/m^3^ × exposure months]); WHO 1.5 level (1.5 to <2 × [25 μg/m^3^ × exposure months]); and WHO 2.0 level (≥2 × 25 μg/m^3^ × exposure months).

### 2.3. Study Population

**Figure 1:**
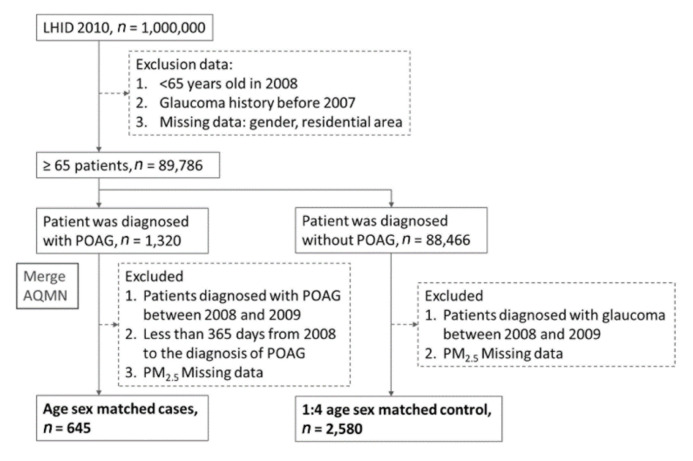
Flowchart of this study population.

This research obtained patient exposure and basic population data from 2008 to 2013 through LHID2010 and AQMN. We recruited individuals aged 65 years or older with no history of glaucoma (ICD9: 365.x) before 2008, and excluded patients with values missing from LHID2010 (Figure 1). From 2008–2013, 1320 patients were diagnosed with POAG (ICD9: 365.1x), whereas 88,466 patients were diagnosed as having non-glaucoma (ICD9: 365.x). In this nested case–control study, we excluded from the case group patients diagnosed with POAG from 2008 to 2009 but with less than 365 days from the start of 2008 to the diagnosis of POAG, while patients from the control group were excluded if they were diagnosed with any other type of glaucoma from 2008 to 2009. Patients for whom PM_2.5_ data were missing were excluded from both groups. After matching for age, gender, and the endpoint of observations, the case group included 645 patients, and the control group included 2580 patients.

### 2.4. Comorbidities

This study used the following comorbidities [24,25] as confounding factors for regression adjustment: hypertension (ICD9: 401.x-405.x), ischemic heart disease (ICD9: 410.x-414.x), hyperlipidemia (ICD9: 272.0–272.4), congestive heart failure (ICD9: 428.x), peripheral vascular disease (ICD9: 433.x), atrial fibrillation (ICD9: 427.31), ischemic stroke (ICD9: 434.11), headaches (ICD9: 784), migraines (ICD9: 346), epilepsy and recurrent (ICD9: 345), dementia (ICD9: 290), rheumatoid arthritis (ICD9: 714.0), systemic lupus erythematosus (ICD9: 710.0), diabetes (ICD9: 250.x), asthma (ICD9: 493), chronic kidney disease (ICD9: 585), hepatitis B (ICD9: 070.2, 070.3, V02.61), fluid, electrolyte, acid–base disorders (ICD9: 276.x), tuberculosis (ICD9: 010.x - 017.x.), anemia (ICD9: 280.x-285.x), peptic ulcer (ICD9: 533), depression (ICD9: 311), and malignant disease (ICD9: 14x-23x).

### 2.5. Statistical Analysis

We used the χ^2^ test to assess differences between the case and control groups for categorical variables and two-tailed *t* tests to determine between-group differences in continuous variables. Logistic regression emphasizes the independence of irrelevant alternatives [26,27]. We employed both univariate and multivariate logistic regression to estimate the odds ratios (ORs) and their 95% CIs of risk factors for POAG. The adjusted variables include gender, age, low income (no, yes), urbanization level [28] (highly urbanized, moderate urbanization, emerging town, general town, aged township, agricultural town, remote township), and comorbidity.

P(Y = 1|X_1_...X_k_) = 1/(1 + e^− [a + b1 × 1 + b2 × 2 + … + bk × k]^). (1)

The purpose of this study was to illustrate the risk assessment of risk factors for POAG populations. Given number-independent observations, we fit a logistic regression model equation given Equation (1). We estimated the values of the unknown parameters a, b1, b2,..., bk, to get more accurate results after mutual interference. All statistical analyses were performed using SAS v. 9.3 (SAS Institute, Cary, NC, USA), with *P*  <  0.05 considered statistically significant..

## 3. Results

### 3.1. Patient Characteristics

Table 1 presents the basic characteristics of case group (POAG) and control group (non-POAG) patients with PM_2.5_ concentration exposure. After matching age and gender, patients over 65 years of age from 2008 to 2013 were observed from samples of Taiwan LHID2010. There were no significant differences in gender, age, or low income between case and control groups. Significant differences were observed for urbanization level (*P* < 0.05). A higher proportion of case group patients were highly urbanized (30.7%), compared with a higher proportion of moderate urbanization for control patients (25.85%). Among the comorbidities, there were significant between-group differences for hypertension, ischemic heart disease, hyperlipidemia, peripheral artery disease, atrial fibrillation, headaches, diabetes, anemia, peptic ulcer, and malignant disease, respectively. The proportions of persons with these conditions were higher for case group patients (69.61%, 22.64%, 48.53%, 8.68%, 5.27%, 21.24%, 35.04%, 15.81%, 10.23%, and 16.28%, respectively) than for control patients (62.64%, 17.52%, 34.07%, 6.09%, 2.48%, 16.98%, 26.71%, 10.62%, 6.59%, and 12.02%, respectively).

### 3.2. OR of PM_2.5_ Exposure as Risk Factor for POAG by Logistic Regression

Table 2 expresses the distributions of WHO PM_2.5_ levels for case group patients and controls. Among all participants, the median PM_2.5_ level was 1159.84 μg/m^3^ and the mean [SD] was 1262.18 [629.57] μg/m^3^, the mean [SD] of follow up months was 48.05 [15.07]. At the PM_2.5_ normal level, there were 210 controls and 56 case group patients (median PM_2.5_ = 691.3 μg/m^3^, mean PM_2.5_ [SD] = 688.4 [283.57] μg/m^3^, mean follow up months [SD] was 43.30 [15.07]). The WHO 1.0 level contained the highest proportion of participants, with 1457 controls and 318 case group patients (median = 1046.42 μg/m^3^, mean [SD] = 1080.31 [469.86] μg/m^3^, mean follow up months [SD] was 42.90 [15.08]). The WHO 1.5 level contained 797 controls and 209 case group patients (median = 1478.70 μg/m^3^, mean [SD] 1569.97 [680.75] μg/m^3^, mean follow up months [SD] was 52.00 [13.93]), whereas the WHO 2.0 level contained 116 controls and 62 case group patients (median = 2221.87 μg/m^3^, mean [SD] = 2193.6 [133.81] μg/m^3^, mean follow up months [SD] was 53.56 [11.83]). The odds ratio of per WHO standard level increase was 1.248 (95% CI 1.106–1.408) using univariate logistic regression, and 1.193 (95% CI 1.050–1.356) with multivariate logistic regression. Compared with the normal level, the OR of the WHO 2.0 level was 2.004 (95% CI 1.308–3.071, *P* < 0.05) through univariate logistic regression and 1.668 (95% CI 1.045–2.663, *P* < 0.05) through multivariate logistic regression. These data indicate that as the PM_2.5_ level rises, POAG risk increases, and it is significant at the WHO 2.0 level.

Table 3 expresses the risks of confounding variables that were used to adjust the PM_2.5_ level for POAG. With multivariate logistic regression, the odds ratio of low income was 0.744 (95% CI 0.603–0.919, *P* < 0.05). For urbanization level, compared with high urbanization, the calculated ORs were as follows: for emerging town, 0.640 (95% CI 0.469–0.872, *P* < 0.05); for general town, 0.628 (95% CI, 0.464–0.852, *P*< 0.05); for aged township, 0.545 (95% CI, 0.320–0.927, *P*< 0.05); and for remote township, 0.413 (95% CI 0.254–0.669, *P* <.05). For comorbidities, the calculated ORs were as follows: for hyperlipidemia, 1.588 (95% CI 1.305–1.932, *P* < 0.05); for atrial fibrillation, 2.088 (95% CI 1.324–3.292, *P* < 0.05); for anemia, 1.410 (95% CI 1.085–1.833, *P* < 0.05); and for depression, 1.577 (95% CI 1.157–2.149, *P* < 0.05).

### 3.3. ORs of PM_2.5_ Level As a Risk Factor for POAG in Subgroups.

Table 4 shows the risk of PM_2.5_ WHO 2.0 level for POAG in each subgroup. Compared with the normal level, the OR of WHO 2.0 level was 2.148 (95% CI 1.100–4.194, *P* < 0.05) and *P*_trend_ = 0.0231 in non-hyperlipidemia patients; for non-atrial fibrillation patients, 1.673 (95% CI 1.037–2.699, *P* < 0.05) and *P*_trend_ = 0.0120; for non-anemia patients, 1.814 (95% CI 1.092–3.012, *P* < 0.05) and *P*_trend_ = 0.0062; and for non-depression patients, 1.651 (95% CI 1.012–2.692, *P* < 0.05). After systematically excluding these confounding factors, we determined that increased PM_2.5_ exposure is related to the risk of POAG, especially at the WHO 2.0 level.

## 4. Discussion

In this study, we identified an association between PM_2.5_ exposure and increased POAG risk in elderly patients. More importantly, the increased prevalence of POAG was based on exposure to higher levels of PM_2.5_. These findings support existing evidence concerning the association between air pollution and POAG with comorbidity, suggesting that PM_2.5_ exposure increases POAG risk.

Glaucoma is attributable to external factors including environmental risk and socio-economic development [29,30]; internal factors such as age, family history, ethnicity, and high intraocular pressure (IOP) may cause glaucoma development [29,31]. Our results indicate that the proportion of people with POAG in moderate and highly-urbanized settings was higher than in more outlying regions (e.g., agricultural towns and remote townships). A study illustrated that areas with high PM_2.5_ concentrations are distributed among highly-developed cities in Eastern China [32]. The WHO indicated that people living in low- and middle-income countries experience the burden of 90% of outdoor air pollution, mostly in the Southeast Asia and Western Pacific regions [33,34]. For example, the Beijing–Tianjin–Hebei region is the largest urban agglomeration in China; Beijing’s annual average PM_2.5_ concentration varies from 87.6 μg/m^3^ to 111.9 μg/m^3^ [35]. Several studies have indicated that PM_2.5_ concentrations in urban areas are substantially higher than in rural areas in China [36]. The outdoor PM_2.5_ components come mainly from carbonaceous residues of combustion-powered motor vehicles, population activities, and forest coverage in the USA [37]. These studies confirm that highly polluted areas are mainly concentrated in highly urbanized settings that produce aggregated effects of PM_2.5_.

The logistic regression analysis of PM_2.5_ level and POAG showed that patients exposed to the WHO 2.0 level of PM_2.5_ are about 68.8% more likely than controls to develop POAG (OR 1.668, 95% CI 1.045–2.663). In addition, per WHO standard level increases may also increase the risk of POAG (OR 1.193, 95% CI 1.050–1.356). Our results suggest that PM_2.5_ concentration is a key parameter in associations between PM_2.5_ exposure and human health. Regions with higher PM_2.5_ concentrations more strongly deteriorate population health [38,39]. Previous studies have shown that oxidative stress and inflammation are potentially important indicators of disease caused by particles from traffic, industrial, and other urban sources (e.g., regions of high PM_2.5_ concentration) [7,40]. Recent evidence from the Rome Longitudinal Study indicated a 4% increased risk of all-cause mortality per 10 μg/m^3^ increase in PM_2.5_ exposure, with higher associated risks for ischemic heart disease mortality and lung cancer [41,42].

Glaucoma is an increasingly prevalent public health concern that is the second leading cause of blindness worldwide [43]. Many investigations indicate that open-angle glaucoma is caused by occluding blood flow to the optic nerve head [44]. In this study, we found that individuals with hyperlipidemia, atrial fibrillation, anemia, and peptic ulcers living under high PM_2.5_ conditions are at increased risk of developing POAG. Wang et al. reported that both hyperlipidemia and hypertriglyceridemia were significantly associated with glaucoma [45]. A previous study using the National Health Insurance Database demonstrated that patients with hyperlipidemia in Taiwan population had a 1.11-fold increased POAG risk [25,46]. A previous study also indicated that elderly patients with atrial fibrillation may be at strong risk for normal tension glaucoma [47]. An association between atrial fibrillation and posterior ciliary vessel occlusion that may cause sudden ischemia of the optic nerve was previously documented [48,49,50]. Iron deficiency anemia, which results in imbalance between oxidants and antioxidants, can affect the entire nervous system, including the optic nerve [51]. However, DeMaman et al. found no significant difference in frequency of iron deficiency anemia between glaucoma and control groups [51]. Peptic ulcers were significantly associated with increasing primary angle closure glaucoma (PACG) risk in Taiwan population [46]. In a previous study, it was shown that exposure to high levels of PM_2.5_ caused the ganglion cell–inner plexiform layer to be more vulnerable and a decrease in thickness resulted in the higher risk of glaucoma [52]. For short- and long-term exposure, the risk of glaucoma was induced by PM10 in childhood [53]. Additionally, high levels of air pollution, being older age or female, and experiencing higher ambient ultraviolet radiation had already been reported to be significantly associated with the higher burden of glaucoma [29]. The results from the present study are similar to prior studies. Furthermore, we firstly proposed that the prolonged exposure level of PM_2.5_ has significant positive correlation with POAG risk excluding comorbidity in Taiwan. Based on these findings, we could suggest that the exposure level of PM_2.5_ is an independent risk of POAG.

In the POAG patient eyes, the rate of vision field loss in the first half is faster than that in the second half, especially in the central, paracentral, and peripheral arcuate 2 regions. The pathological exchange was not found in the PACG patient eyes [54]. Transforming growth factor-β2 (TGFβ2) and secreted frizzled-related protein-1 (SFRP1) levels were detected in aqueous humor levels (AH) samples from different glaucoma patients. It was found that (1) the concentration of TGFβ2 in AH of POAG patients was higher; (2) angle-closure glaucoma patients with higher IOP had higher levels of cytokines; (3) there were negative correlations between SFRP1 and IOP in the POAG patients [55]. The evidence shows that PM_2.5_ may affect the development of POAG after affecting TGFβ generation, but further studies are needed to confirm this. There are several approaches for preventing the risk of PM_2.5_ via reduction of outdoor and indoor exposure, using air cleaners and air filtration masks [56]. Regular physical exercises, eating more vegetables, improvement in the quality of life, and upregulation of the connection of the nervous system all reduce the risk of glaucoma [57].

This study has several potential limitations. First, laboratory data were lacking; the patients’ biochemical markers, such as blood sugar and blood lipid values, could not be collected. However, after adjustments for the confounding factors of comorbidities, no differences were noted in the impact of this restriction on our results. Second is the accuracy of the patients’ PM_2.5_ exposure values. Because the patients’ residence district was used as the basis for PM_2.5_ observation, it was uncertain whether the patient had moved to other areas (and how much time was spent there), resulting in some errors in the observations of PM_2.5_. However, for exposure over a long term, a patient’s main activity area should be based on their residence location, so this restriction did not affect our results strongly. Third, the details of the patients’ usual lifestyle habits, such as smoking and drinking, were not recorded in the health insurance database; therefore, how our results might have been influenced by these factors remains difficult to understand.

In summary, our results provide potential explanations concerning the strong relationship between populations exposed to high PM_2.5_ levels and open-angle glaucoma without comorbidity. In this study, PM_2.5_ was an independent factor associated with POAG. Future work further illuminating the mechanisms connecting PM_2.5_ exposure and open-angle glaucoma is warranted.

## Figures and Tables

**Table 1:** Baseline characteristics of participants of primary open-angle glaucoma (POAG) and comparison.

		Comparison		POAG		*P*-value
		(*n* = 2580)		(*n* = 645)		
Gender						
	Female	1228	(47.6%)	307	(47.6%)	1.0000
	Male	1352	(52.4%)	338	(52.4%)	
Age						
	Mean ± SD	72.64 ± 5.58		72.57 ± 5.66		0.7915
Low income						
	Yes	1620	(62.79%)	405	(62.79%)	1.0000
	No	960	(37.21%)	240	(37.21%)	
Urbanization level						
	Highly urbanized	563	(21.82%)	198	(30.7%)	<.0001
	Moderate urbanization	667	(25.85%)	180	(27.91%)	
	Emerging town	402	(15.58%)	79	(12.25%)	
	General town	489	(18.95%)	105	(16.28%)	
	Aged Township	101	(3.91%)	21	(3.26%)	
	Agricultural town	195	(7.56%)	37	(5.74%)	
	Remote township	163	(6.32%)	25	(3.88%)	
Comorbidity						
	Hypertension	1616	(62.64%)	449	(69.61%)	0.0010
	Ischemic heart disease	452	(17.52%)	146	(22.64%)	0.0048
	Hyperlipidemia	879	(34.07%)	313	(48.53%)	<.0001
	Conge stive heart failure	256	(9.92%)	77	(11.94%)	0.1523
	Peripheral vascular disease	157	(6.09%)	56	(8.68%)	0.0002
	Atrial fibrillation	64	(2.48%)	34	(5.27%)	0.0028
	Ischemic stroke	85	(3.29%)	19	(2.95%)	0.6539
	Headaches	438	(16.98%)	137	(21.24%)	0.0047
	Migraines	75	(2.91%)	29	(4.5%)	0.0716
	Epilepsy and recurrent	41	(1.59%)	16	(2.48%)	0.1775
	Dementia	173	(6.71%)	44	(6.82%)	0.9161
	Rheumatoid arthritis	82	(3.18%)	24	(3.72%)	0.5093
	Systemic lupus erythematosus	3	(0.12%)	2	(0.31%)	0.3979
	Diabetes	689	(26.71%)	226	(35.04%)	<.0001
	Asthma	622	(24.11%)	161	(24.96%)	0.6516
	Chronic kidney disease	131	(5.08%)	39	(6.05%)	0.3489
	Hepatitis B	39	(1.51%)	12	(1.86%)	0.5505
	Fluid, Electrolyte, Acid–Base Disorders	55	(2.13%)	23	(3.57%)	0.0678
	Tuberculosis	63	(2.44%)	16	(2.48%)	0.9546
	Anemia	274	(10.62%)	102	(15.81%)	0.0009
	Peptic ulcer	170	(6.59%)	66	(10.23%)	0.0049
	Depression	52	(2.02%)	16	(2.48%)	0.4893
	Malignant disease	310	(12.02%)	105	(16.28%)	0.0074

Abbreviation: POAG: primary open-angle glaucoma; SD: standard deviation.

**Table 2:** Logistic regression analysis of particulate matter (PM)_2.5_ level and POAG.

	*n* (%)		Distribution of PM_2.5_ (μg/m^3^)			Odd ratio (95% CI)	
	Comparison	POAG	Median	Mean (SD)	Follow up Months Mean (SD)	Univariate	Multivariate
Total participants							
Per WHO standard level increase	2580	645	1159.84	1262.18 (629.57)	48.05 (15.07)	1.248 (1.106–1.408)	1.193 (1.050–1.356)
PM_2.5_ WHO standard level (reference: normal standard)							
Normal level	210 (8.14%)	56 (8.56%)	691.30	688.4 (283.57)	43.30 (16.05)	Reference	Reference
WHO 1.0 level	1457 (56.47%)	318 (48.62%)	1046.42	1080.31 (469.86)	42.90 (15.08)	0.818 (0.595–1.126)	0.825 (0.588–1.158)
WHO 1.5 level	797 (30.89%)	209 (31.96%)	1478.70	1569.97 (680.75)	52.00 (13.93)	0.983 (0.706–1.370)	0.982 (0.690–1.396)
WHO 2.0 level	116 (4.5%)	62 (9.48%)	2221.87	2193.60 (133.81)	53.56 (11.83)	2.004 (1.308–3.071)	1.668 (1.045–2.663)

Abbreviation: CI: confidence interval; POAG: primary open-angle glaucoma; SD: standard deviation; WHO: World Health Organization. Adjusted for gender, age, low income, urbanization level, and comorbidity.

**Table 3:** Logistic regression analysis of PM_2.5_ level and POAG.

		Odd ratio (95% CI)	
		Univariate	Multivariate
PM_2.5_ WHO standard level (reference: normal level)			
	WHO 1.0 level	0.818 (0.595–1.126)	0.825 (0.588–1.158)
	WHO 1.5 level	0.983 (0.706–1.370)	0.982 (0.690–1.396)
	WHO 2.0 level	2.004 (1.308–3.071)	1.668 (1.045–2.663)
Gender (reference: female)			
	Male	1.000 (0.841–1.189)	1.093 (0.911–1.311)
Age (reference:general population)			
	Per year	0.998 (0.983–1.013)	0.999 (0.982–1.015)
Low income (reference: no)			
	yes	1.000 (0.837–1.195)	0.744 (0.603–0.919)
Urbanization level (reference: highly urbanized)			
	Moderate urbanization	0.767 (0.609–0.967)	0.822 (0.642–1.053)
	Emerging town	0.559 (0.418–0.747)	0.640 (0.469–0.872)
	General town	0.611 (0.468–0.796)	0.628 (0.464–0.852)
	Aged Township	0.591 (0.360–0.972)	0.545 (0.320–0.927)
	Agricultural town	0.540 (0.366–0.794)	0.471 (0.307–0.723)
	Remote township	0.436 (0.278–0.685)	0.413 (0.254–0.669)
Comorbidity (reference: no)			
	Hypertension	1.366 (1.135–1.645)	1.074 (0.873–1.320)
	Ischemic heart disease	1.378 (1.116–1.700)	1.090 (0.865–1.374)
	Hyperlipidemia	1.824 (1.532–2.172)	1.588 (1.305–1.932)
	Congestive heart failure	1.231 (0.939–1.613)	1.015 (0.752–1.371)
	Peripheral vascular disease	1.708 (1.304–2.236)	1.393 (0.998–1.943)
	Atrial fibrillation	2.188 (1.430–3.347)	2.088 (1.324–3.292)
	Ischemic stroke	0.891 (0.538–1.476)	0.796 (0.471–1.347)
	Headaches	1.319 (1.064–1.635)	1.204 (0.954–1.519)
	Migraines	1.573 (1.016–2.437)	1.417 (0.891–2.253)
	Epilepsy and recurrent	1.576 (0.879–2.827)	1.635 (0.891–3.002)
	Dementia	1.019 (0.723–1.435)	0.902 (0.625–1.301)
	Rheumatoid arthritis	1.177 (0.741–1.871)	1.031 (0.635–1.675)
	Systemic lupus erythematosus	2.672 (0.446–16.023)	3.126 (0.468–20.892)
	Diabetes	1.481 (1.232–1.779)	1.175 (0.958–1.440)
	Asthma	1.047 (0.857–1.279)	0.934 (0.755–1.155)
	Chronic kidney disease	1.203 (0.832–1.739)	0.943 (0.638–1.394)
	Hepatitis B	1.235 (0.643–2.373)	0.993 (0.502–1.963)
	Fluid, Electrolyte, Acid–Base Disorders	1.698 (1.035–2.784)	1.455 (0.866–2.446)
	Tuberculosis	1.016 (0.583–1.771)	1.004 (0.562–1.793)
	Anemia	1.581 (1.236–2.021)	1.410 (1.085–1.833)
	Peptic ulcer	1.616 (1.199–2.178)	1.577 (1.157–2.149)
	Depression	1.237 (0.701–2.180)	1.025 (0.568–1.850)
	Malignant disease	1.424 (1.120–1.811)	1.280 (0.995–1.646)

Abbreviation: CI: confidence interval. Adjusted for gender, age, low income, urbanization level, and comorbidity.

**Table 4:** Logistic regression analysis of PM_2.5_ level and POAG in subgroups.

	Odd Ratio (95% CI), Reference: Normal Level			*P*_trend_
	WHO 1.0 level	WHO 1.5 level	WHO 2.0 level	
Total	0.825 (0.588–1.158)	0.982 (0.690–1.396)	1.668 (1.045–2.663)	0.0068
Hyperlipidemia				
Yes	0.678 (0.419–1.097)	0.876 (0.532–1.441)	1.291 (0.661–2.521)	0.1567
No	0.999 (0.609–1.639)	1.122 (0.670–1.880)	2.148 (1.100–4.194)	0.0231
Atrial fibrillation				
Yes	0.474 (0.025–8.961)	2.163 (0.108–43.256)	0.196 (0.004–9.417)	0.9929
No	0.818 (0.58–1.155)	0.957 (0.67–1.369)	1.673 (1.037–2.699)	0.0120
Anemia				
Yes	0.640 (0.262–1.566)	0.559 (0.210–1.489)	0.843 (0.223–3.193)	0.5377
No	0.828 (0.571–1.202)	1.021 (0.695–1.500)	1.814 (1.092–3.012)	0.0062
Peptic ulcer				
Yes	0.877 (0.243–3.166)	0.777 (0.214–2.814)	5.602 (0.597–52.587)	0.6761
No	0.832 (0.581–1.191)	1.014 (0.698–1.471)	1.651 (1.012–2.692)	0.0028

Abbreviation: CI: confidence interval. Adjusted for gender, age, low income, urbanization level, and comorbidity.

## Data Availability

The data are not publicly available due to privacy or ethical restrictions.
